# Ribosomal RNA-Specific Antisense DNA and Double-Stranded DNA Trigger rRNA Biogenesis and Insecticidal Effects on the Insect Pest *Coccus hesperidum*

**DOI:** 10.3390/ijms26157530

**Published:** 2025-08-04

**Authors:** Vol Oberemok, Nikita Gal’chinsky, Ilya Novikov, Alexander Sharmagiy, Ekaterina Yatskova, Ekaterina Laikova, Yuri Plugatar

**Affiliations:** 1 Department of General Biology and Genetics, Institute of Biochemical Technologies, Ecology and Pharmacy, V.I. Vernadsky Crimean Federal University, Simferopol 295007, Russia; voloberemok@gmail.com (V.O.); i.nowikow2012@mail.ru (I.N.); botan_icus@mail.ru (E.L.); 2 Laboratory of Entomology and Phytopathology, Dendrology and Landscape Architecture, Nikita Botanical Gardens—National Scientific Centre of the Russian Academy of Sciences, Yalta 298648, Russia; alexander_sharma@mail.ru (A.S.); vercful@mail.ru (E.Y.); 3 Department of Natural Ecosystems, Nikita Botanical Gardens—National Scientific Centre of the Russian Academy of Sciences, Yalta 298648, Russia; plugatar.y@gmail.com

**Keywords:** contact unmodified antisense DNA biotechnology (CUADb), DNA containment mechanism (DNAc), DNA-programmable plant protection, genetic zipper method, oligonucleotide insecticides, kinase disaster

## Abstract

Contact unmodified antisense DNA biotechnology (CUADb), developed in 2008, employs short antisense DNA oligonucleotides (oligos) as a novel approach to insect pest control. These oligonucleotide-based insecticides target pest mature rRNAs and/or pre-rRNAs and have demonstrated high insecticidal efficacy, particularly against sap-feeding insect pests, which are key vectors of plant DNA viruses and among the most economically damaging herbivorous insects. To further explore the potential of CUADb, this study evaluated the insecticidal efficacy of short 11-mer antisense DNA oligos against *Coccus hesperidum*, in comparison with long 56-mer single-stranded and double-stranded DNA sequences. The short oligos exhibited higher insecticidal activity. By day 9, the highest mortality rate (97.66 ± 4.04%) was recorded in the Coccus-11 group, while the most effective long sequence was the double-stranded DNA in the dsCoccus-56 group (77.09 ± 6.24%). This study also describes the architecture of the DNA containment (DNAc) mechanism, highlighting the intricate interactions between rRNAs and various types of DNA oligos. During DNAc, the Coccus-11 treatment induced enhanced ribosome biogenesis and ATP production through a metabolic shift from carbohydrates to lipid-based energy synthesis. However, this ultimately led to a ‘kinase disaster’ due to widespread kinase downregulation resulting from insufficient ATP levels. All DNA oligos with high or moderate complementarity to target rRNA initiated hypercompensation, but subsequent substantial rRNA degradation and insect mortality occurred only when the oligo sequence perfectly matched the rRNA. Both short and long oligonucleotide insecticide treatments led to a 3.75–4.25-fold decrease in rRNA levels following hypercompensation, which was likely mediated by a DNA-guided rRNase, such as RNase H1, while crucial enzymes of RNAi (DICER1, Argonaute 2, and DROSHA) were downregulated, indicating fundamental difference in molecular mechanisms of DNAc and RNAi. Consistently, significant upregulation of RNase H1 was detected in the Coccus-11 treatment group. In contrast, treatment with random DNA oligos resulted in only a 2–3-fold rRNA decrease, consistent with the normal rRNA half-life maintained by general ribonucleases. These findings reveal a fundamental new mechanism of rRNA regulation via complementary binding between exogenous unmodified antisense DNA and cellular rRNA. From a practical perspective, this minimalist approach, applying short antisense DNA dissolved in water, offers an effective, eco-friendly and innovative solution for managing sternorrhynchans and other insect pests. The results introduce a promising new concept in crop protection: DNA-programmable insect pest control.

## 1. Introduction

Hemipterans, particularly sternorrhynchans, are recognized as major pests of numerous agricultural crops. They are primary vectors for plant DNA viruses and bacteria and are responsible for substantial yield losses worldwide [1,2]. Currently, these pests are primarily managed using neonicotinoid insecticides. However, intensive use has led to the emergence of resistance in field populations [3,4,5,6]. Moreover, hemipterans excrete honeydew contaminated with neonicotinoids, which may have lethal and sublethal effects on beneficial insects at various concentrations [7]. The rise of insecticide resistance has prompted the continuous search for novel and effective pest control strategies [8,9]. The historical reliance on chemical insecticides underscores the urgent need for a paradigm shift in pest management, toward the development of control agents with long-term efficacy and minimal ecological impact.

In 2008, a new dimension of insect pest control—DNA-programmable plant protection—was introduced when unmodified DNA was found to exhibit insecticidal activity [10]. Since then, significant progress has been made in this field by our team, including the identification of ribosomal RNAs as optimal targets, elucidation of the underlying mechanism (DNA containment or DNAc mechanism), and identification of highly susceptible insect groups, especially sternorrhynchans [11]. For the first time, it was shown that short unmodified antisense DNA can both upregulate and, subsequently, downregulate rRNA genes expression, which governs 80% of the cell’s total RNA. This dynamic regulation of rRNA may play a key role in rDNA transcription, the replication of DNA viruses, and cellular antiviral defenses [12].

Over recent years, contact-unmodified antisense DNA biotechnology (CUADb) has emerged as a powerful and selective approach to pest control. This technology utilizes short antisense oligonucleotides (olinscides or DNA insecticides) and operates through the so-called ‘genetic zipper’ mechanism, where the olinscide binds its complementary mature rRNA and/or pre-rRNA target and, in the presence of DNA-guided rRNases (such as RNase H1), suppresses gene expression [13]. The CUADb approach has proven effective against a wide range of hemipteran pests, including soft and armored scale insects, whiteflies, psyllids, mealybugs, and aphids, as well as other pest groups such as thrips and spider mites. A recent opinion article in *Frontiers in Agronomy* summarized a successful list of pests targeted by oligonucleotide insecticides [14]. Remarkably, all 13 tested olinscides were selected using a single algorithm and demonstrated high efficacy, causing on average 80.04 ± 12.73% mortality in sternorrhynchans within 3–14 days after one or two applications. Due to its simplicity and effectiveness, the CUADb-based ‘genetic zipper’ method offers a promising alternative to RNA interference (RNAi) and CRISPR/Cas9 systems, both of which require more complex design and optimization [15].

Unlike RNAi and CRISPR/Cas9, which were originally developed as laboratory tools and only later investigated for their potential use in pest control, CUADb was specifically developed for insect pests and has recently been shown to play a fundamental role in rRNA biogenesis [11,14,16,17,18]. While RNAi and CRISPR/Cas9 offer valuable molecular tools, they lack streamlined protocols for large pest control applications, often requiring case-by-case optimization using trial-and-error approaches [19,20].

Ribosomal RNAs represent ideal targets for oligonucleotide insecticides due to their abundance, accounting for 80% of total cellular RNA, and their essential roles in translation, metabolism, and cellular signaling [21]. In contrast, messenger RNAs (mRNAs), which constitute only about 5% of total RNA, offer limited targeting potential. Targeting mature rRNA and/or pre-rRNA provides a high signal-to-noise ratio (~10^5^:1) compared to mRNA [22]. Although exceptions exist, such as when specific genes like IAPs are highly expressed during viral infection [23], rRNA remains the most reliable target. Oligonucleotide insecticides function through the **DNA containment mechanism (DNAc)**, a two-step process identified in sternorrhynchans [17,18]. This process occurs primarily in the nucleus—specifically, the nucleolus—where ribosome biogenesis takes place [24]. In the first step, the target rRNA is functionally ‘arrested,’ leading to its hypercompensation. In the second step, the target rRNA undergoes degradation mediated by DNA-guided rRNases such as RNase H1 [18]. Formation of a duplex between the olinscide and rRNA mimics a zipper mechanism, effectively shutting down rRNA expression and resulting in pest death [13].

The high efficacy of oligonucleotide insecticides against sternorrhynchans may partly be attributed to anatomical features that facilitate the uptake of DNA oligos, such as spiracular pores and other surface structures [25]. As next-generation insecticides, these contact-active agents are biodegradable and selective, with a low carbon footprint, and are less prone to resistance development [18,26]. Their minimalist composition, short antisense DNA dissolved in water, minimizes environmental risk. Moreover, if resistance does emerge, new olinscides can be easily designed by shifting the target sequence upstream or downstream from the resistance-conferring site [13,18,27]. Importantly, the **3′-end complementarity rule** is critical for ensuring maximum insecticidal efficacy [18]. Additionally, non-canonical base pairing, such as A:C mismatches or G_olinscide_–U_rRNA_ interactions, should be considered during oligonucleotide design [18,28,29]. Liquid-phase DNA synthesis using phosphoramidite chemistry enables large-scale and cost-effective production of these molecules [30,31], reducing the cost of application to as low as USD 0.50 per hectare in aphid control [15]. Thus, oligonucleotide insecticides are not only environmentally safe but also economically competitive with traditional chemical insecticides [32,33,34].

Our research group pioneered the use of contact antisense DNA for plant protection in 2008 [10], re-evaluated the concept in 2019 [14], and continues to optimize CUADb formulations. Oligonucleotide insecticides are highly effective against Hemiptera [35] and moderately effective against Lepidoptera [23,36] and Coleoptera [37]. According to recent estimates, the ‘genetic zipper’ method has the potential to manage 10–15% of the world’s most destructive insect pests [15]. Furthermore, this approach is being extended to additional pest groups [11,38,39] and enables the development of species-specific mixed formulations targeting complex pest communities [18]. The addition of auxiliary formulation components, such as spreaders, adhesives, penetrators, or UV protectants, can be tailored for each application, but must be evaluated for both efficacy and safety. Oligonucleotide insecticides are also compatible with viral [23] and fungal [38] biopesticides, often enhancing pest mortality. It was found that contact delivery of unmodified antisense oligodeoxyribonucleotides (CUADs) is much more efficient than oral delivery of unmodified antisense oligodeoxyribonucleotides (ODUADs) because of active DNases present in the digestive tract of insects [40]. Notably, recent findings have demonstrated the high specificity of oligonucleotide insecticides [36,41] and their safety for a variety of non-target organisms, including *Quercus robur, Malus domestica* [42], *Triticum aestivum* [43,44], and several insects such as *Manduca sexta, Agrotis ipsilon* [45], and *Galleria mellonella* [36].

In this study, we used the brown soft scale insect, *Coccus hesperidum* L. (Hemiptera: Coccidae), as a model system. This species is among the most polyphagous insects, having been recorded on host plants across 345 genera from 121 families. Likely native to South Africa, *C. hesperidum* has spread globally through trade in infested plant material. It feeds on phloem sap and produces honeydew, which promotes the growth of sooty mold on plant surfaces. This indirectly damages plants by blocking light and gas exchange, reducing photosynthesis and overall plant vigor. Though widespread and not listed under plant quarantine, *C. hesperidum* remains a key pest of greenhouse crops and citrus and has an exceptionally broad range of host plants, including olive, avocado, cotton, mango, cocoa, fig, hibiscus, oleander, palm, fern, and orchid [46]. In this research, we evaluated the insecticidal efficacy and cellular effects of short (11-mer) and long (56-mer) DNA fragments—both single- and double-stranded—designed with perfect or partial complementarity to the 28S rRNA of *C. hesperidum*. Our primary objective was to assess the performance of these sequences and to deepen our understanding of DNAc mechanism within insect cells.

## 2. Results

### 2.1. Mortality of C. hesperidum After Treatment with Single-Stranded and Double-Stranded DNA Sequences in the Natural Habitat

**Figure 1:**
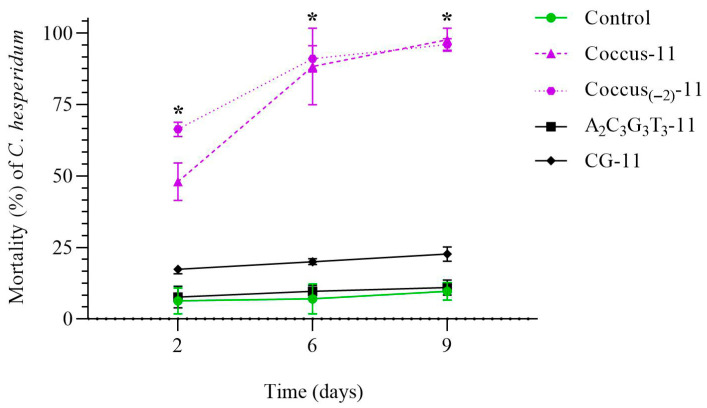
Dynamics of mortality of *C. hesperidum* after contact treatment with water, Coccus-11, Coccus_(−2)_-11, A_2_C_3_G_3_T_3_-11, and CG-11. The significance of differences in the groups of oligonucleotide insecticides (Coccus-11 and Coccus_(−2)-_11) compared to water-treated control is indicated by * at *p* < 0.05.

The insecticidal potential of 11-mer oligonucleotide insecticide Coccus-11 was evaluated based on its effect on the viability of *C. hesperidum* larvae. On the second day, Coccus-11 induced significant pest mortality of 48.08 ± 6.56% (χ^2^ = 145.79, *p* < 0.001, N = 600, df = 1) compared to water-treated control (Figure 1). Similarly, the oligonucleotide insecticide Coccus_(−2)_-11 caused 66.33 ± 2.51% mortality on the second day (χ^2^ = 249.64, *p* < 0.001, N = 600, df = 1) (Figure 1). Mortality in the Coccus-11 treatment group increased progressively over time. By the sixth and ninth days, mortality reached 88.33 ± 13.42% (χ^2^ = 401.61, *p* < 0.001, N = 600, df = 1), and 97.66 ± 4.04% (χ^2^ = 463.62, *p* < 0.001, N = 600, df = 1), respectively. A similar trend was observed for Coccus_(−2)_-11, with mortality rates of 91.11 ± 4.58% (χ^2^ = 430.49, *p* < 0.001) on the sixth and 96.20 ± 2.14% (χ^2^ = 445.19, *p* < 0.001, N = 600, df = 1) on the ninth day. In contrast, the random oligonucleotide A_2_C_3_G_3_T_3_-11 did not exhibit any significant insecticidal effect compared to water-treated control, while CG-11 showed only a weak insecticidal trend. On the ninth day, the mortality rates in the A_2_C_3_G_3_T_3_-11 and CG-11 groups were 10.33 ± 1.53% (χ^2^ = 0.02, *p* > 0.892, N = 600, df = 1) and 22.67 ± 2.52% (χ^2^ = 17.75, *p* < 0.001, N = 600, df = 1), respectively (Figure 1).

**Figure 2:**
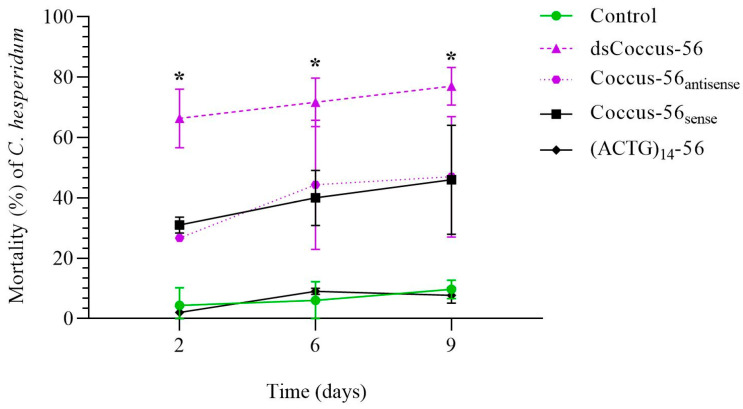
Dynamics of mortality of *C. hesperidum* after contact treatment with water, dsCoccus-56, Coccus-56_antisense_, Coccus-56_sense_, and (ACTG)_14_-56. The significance of differences in the groups of the experiment (dsCoccus-56, Coccus-56_antisense_, Coccus-56_sense_) compared to the water-treated control is indicated by * at *p* < 0.05.

Similar trends were observed for long DNA sequences. The dsCoccus-56 treatment significantly increased pest mortality to 66.33 ± 9.71% on the second day compared to the water-treated control (χ^2^ = 249.64, *p* < 0.001, N= 600, df = 1) (Figure 2). Mortality in Coccus-56_antisense_, Coccus-56_sense_, (ACTG)_14_-56 groups was 26.67 ± 1.15% (χ^2^ = 71.51, *p* < 0.001, N = 600, df = 1), 31.14 ± 2.64% (χ^2^ = 55.43, *p* < 0.001, N = 600, df = 1), and 2.33 ± 1.52% (χ^2^ = 3.87, *p* > 0.05, N = 600, df = 1), respectively.

By the sixth day, all groups except (ACGT)_14_-56 showed significantly higher mortality than the control (dsCoccus-56, χ^2^ = 249.11, *p* < 0.001, N = 600, df = 18; Coccus-56_antisense_, χ^2^ = 96.12, *p* < 0.001, N = 600, df = 1; Coccus-56_sense_, χ^2^ = 100.27, *p* < 0.001, N = 600, df = 1; (ACGT)_14_-56, χ^2^ = 1.53, *p* > 0.215, N = 600, df = 1). On average, 7.33 ± 4.93%, 71.68 ± 8.17%, 44.33 ± 21.38%, 40.05 ± 9.2%, and 9.26 ± 1.43% of insects died on the sixth day in the water-treated control, dsCoccus-56, Coccus-56_antisense_, Coccus-56_sense_, and (ACGT)_14_-56, respectively (Figure 2).

On the ninth day, mortality was significantly higher in all experimental groups compared to water-treated control (except (ACGT)_14_-56 group) and reached 9.68 ± 3.1% in water-treated control, 77.09 ± 6.24% in dsCoccus-56 group (χ^2^ = 250.15, *p* < 0.001, N = 600, df= 1), 47.36 ± 19.97% in Coccus-56_antisense_ group (χ^2^ = 96.78, *p* < 0.001, N = 600, df = 1), 46.42 ± 18.02% in the Coccus-56_sense_ group (χ^2^ = 101.13, *p* < 0.001, N = 600, df = 1), and 7.68 ± 2.51% in (ACGT)_14_-56 group (χ^2^ = 0.52, *p* > 0.469, N = 600, df = 1) (Figure 2).

Both short (Coccus-11, Coccus_(−2)-_11) and long (dsCoccus–56, Coccus-56_antisense_) oligonucleotide insecticides triggered significant pest mortality. In our opinion, the moderate insecticidal potential of Coccus-56_sense_ is explained by its interference with the normal interaction of native 28S rRNA and ribosomal proteins, in the same manner found for antibiotic binding sites [47]. Generally, short olinscides showed greater insecticidal potential in comparison with longer ones. Though olinscide dsCoccus-56 caused substantially higher pest mortality by 39.61 ± 0.91% in comparison with Coccus-56_antisense_ (χ^2^ = 46.69, *p* < 0.001, N = 600, df = 1) and Coccus-56_sense_ (χ^2^ = 43.46, *p* < 0.001, N = 600, df = 1), it was, on average, 21.11 ± 0.07% lower than that of short olinscides Coccus-11 (χ^2^ = 68.42, *p* < 0.001, N = 600, df = 1) and Coccus_(−2)_-11 (χ^2^ = 56.43, *p* < 0.001, N = 600, df = 1) at the end of the experiment.

Notably, the highest pest mortality occurred between the second and sixth days and was observed only for short and long olinscides. Among all tested short and long olinscides, dsCoccus-56 demonstrated the highest insecticidal effect (66.33 ± 9.71% mortality) on *C. hesperidum* on the second day. On the sixth day, short olinscides (Coccus-11 and Coccus_(−2)-_11) significantly increased their insecticidal effect on the pest and surpassed dsCoccus-56 in efficiency (88.33 ± 13.42% and 91.11 ± 4.58% vs. 71.68 ± 8.17% mortality, respectively) (*p* < 0.05). It seems advisable to use formulations of oligonucleotide insecticides against *C. hesperidum* containing both long double-stranded and short single-stranded DNA sequences. This will allow a rapid (in 1–2 days) insecticidal effect to be achieved with double-stranded olinscides, and the remaining pest population will be reduced by short olinscides in a few days after.

### 2.2. Target rRNA Expression of C. hesperidum During DNAc

**Figure 3:**
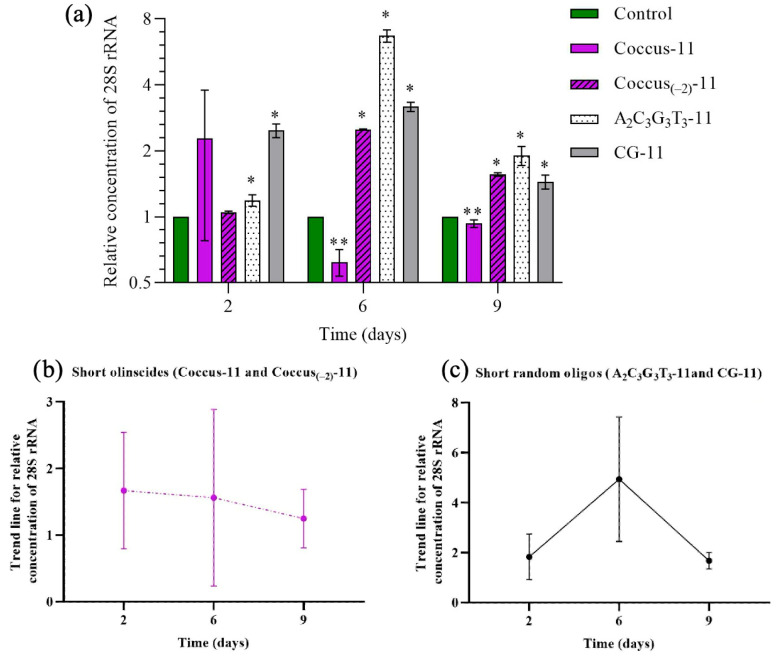
Dynamics of the relative concentration of 28S ribosomal RNA; (**a**) for all short DNA oligos (short olinscides and short random oligos); (**b**) for short olinscides (average concentration); (**c**) short random oligos (average concentration); * is marked when concentration of target 28S rRNA is significantly higher compared to water-treated control (*p* < 0.05); ** is marked when the concentration of 28S rRNA is significantly lower compared to water-treated control (*p* < 0.05); the average relative concentration means that there are individuals in investigated bulk of insects with lower and higher concentrations of target rRNA in comparison with the average number.

**Figure 4:**
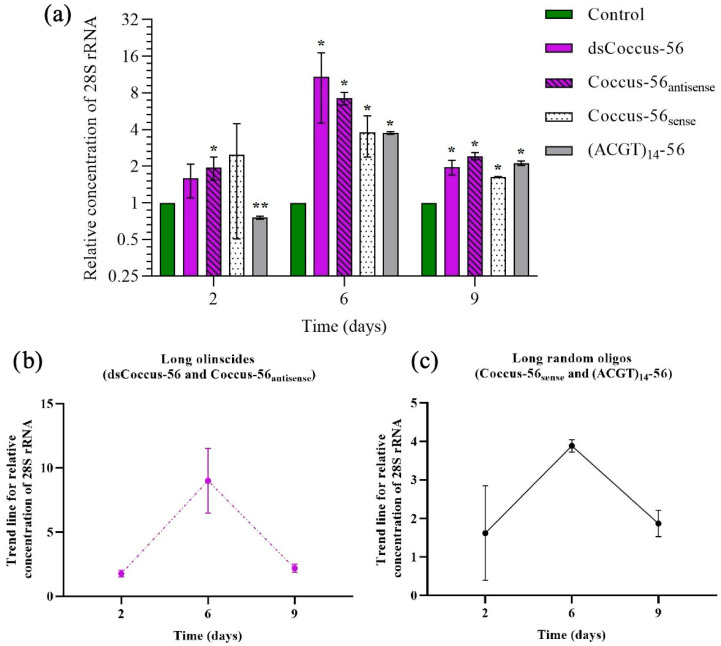
Dynamics of the relative concentration of 28S ribosomal RNA (average concentration) for long olinscides and long random oligos; (**a**) for all long DNA oligos (long olinscides and long random oligos); (**b**) for long olinscides (average concentration); (**c**) long random oligos (average concentration); * is marked when concentration of target 28S rRNA is significantly higher compared to water-treated control (*p* < 0.05); ** is marked when concentration of 28S rRNA is significantly lower compared to water-treated control (*p* < 0.05); average relative concentration means that there are individuals in investigated bulk of insects with lower and higher concentrations of target rRNA in comparison with the average number.

During the DNAc mechanism, hypercompensation of target rRNA was triggered by all DNA oligos (Figure 3a and Figure 4a). Hypercompensation of 28S rRNA progressively increased from the second to the sixth day (the peak for all experimental groups, except Coccus-11, which had a peak on the second day) and then decreased by the ninth day. Generally, short oligos (specific and random) triggered lower levels of rRNA hypercompensation than long oligos. As a trend, among all tested DNA oligos, short oligonucleotide insecticides (Coccus-11 and Coccus_(−2)_-11) triggered the lowest levels of rRNA hypercompensation (Figure 3b) while long olinscides (Coccus56_antisense_ and dsCoccus56) triggered the highest (Figure 4b). Short and long random oligos took an intermediate position (between short and long olinscides) in relation to the level of rRNA hypercompensation (Figure 3c and Figure 4c).

For short olinscides, on average, a 3.75-fold lower concentration of target rRNA was detected on sixth day (the peak of rRNA hypercompensation) compared to short random DNA oligos (Figure 3b,c). In turn, for long olinscides, a 4.25-fold more substantial decrease in target rRNA concentration compared to long random DNA oligos was observed on the ninth day (after the pronounced peak of rRNA hypercompensation on the sixth day) (Figure 4b,c). For both short and long olinscides, the substantial decrease in rRNA concentration is explained by the action of DNA-guided rRNases, such as RNase H1. For long random DNA oligos, the concentration of rRNA decreased by 2.04-fold, and for short random DNA oligos, it decreased by 3.02-fold between the sixth and ninth days, respectively. This corresponds to the normal half-life of rRNAs, which lasts 3–5 days in cells and is degraded by ribonucleases [48].

Coccus-11 and Coccus_(−2)_-11 caused almost similar mortality, but the dynamics of rRNA expression in these two groups differed substantially. Only Coccus-11 downregulated expression of the target rRNA gene by 1.63-fold on the sixth day. This can be explained by the myriad of rRNA binding partners that limit its accessibility to antisense oligonucleotides and the more successful recruitment of DNA-guided rRNase, such as RNase H1 [49,50].

### 2.3. Histological Studies

**Figure 5:**
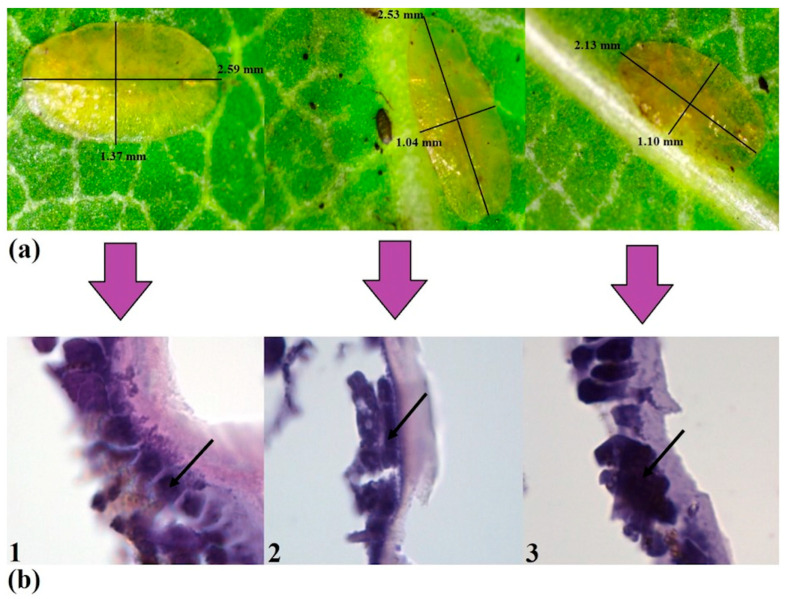
The effect of water (Control), random oligo (A_2_C_3_G_3_T_3_-11), and oligonucleotide insecticide (Coccus-11) on *C. hesperidum* larvae on the second day of the experiment; (**a**) insect morphology was investigated with light microscopy; (**b**) the integument of *C. hesperidum* is represented by a cylindrical single-layer epithelium from different groups of the experiment stained with hematoxylin and eosin; 1—water (Control), 2—random oligo (A_2_C_3_G_3_T_3_-11), 3—oligonucleotide insecticide (Coccus-11); arrows show areas with the most intensive staining by hematoxylin (blue color) in each variant of the experiment.

Histological studies were performed (Figure 5a,b) to detect hypercompensation of rRNA in insect cells caused by DNA oligos. The second day of the experiment with short DNA oligos was selected, during which substantial rRNA hypercompensation was detected in the Coccus-11 group compared to water-treated control. Upon histological examination with hematoxylin and eosin, the apical parts of the insect cells in the control group showed outgrowths. In the recesses between these outgrowths, vesicles of the Golgi apparatus emerged with polysaccharides, forming the cuticle layer by layer. Insects in the control group exhibited a well-defined network of outgrowths, and the layering of the cuticle was also noticeable. From the basement membrane side, the epithelium was washed by hemolymph (Figure 5b—control). Interestingly, in the A_2_C_3_G_3_T_3_-11 group, the color saturation and thickness of the cuticle were similar to those in the water-treated control group (Figure 5b—A_2_C_3_G_3_T_3_-11). Insects in the A_2_C_3_G_3_T_3_-11 group showed minor changes, such as slight thinning of the cuticle layer and a loss of layering.

In the Coccus-11 group, despite a sufficiently tall epithelium and preserved apical processes, the cuticle was substantially thinner than in the control group (Figure 5b—Coccus-11). The cytoplasm of epithelial cells appears denser, with a large number of dark-stained hematoxylin granules. Hematoxylin is known to stain nucleic acids (DNA and RNA), the cell nucleus, ribosomes, and RNA-rich areas of the cytoplasm [51]. Therefore, the intensive staining observed in the Coccus-11 group supports rRNA hypercompensation and an increased level of rRNA biogenesis (confirmed by RT-PCR; Figure 3a), in comparison with the water-treated control and A_2_C_3_G_3_T_3_-11 groups on the second day after the treatment with Coccus-11 (Figure 3a).

### 2.4. Oligonucleotide Insecticides Are Predominantly Contact Insecticides

During the experiment, it was noticed that *C. hesperidum* occurred on the abaxial side of *Pittosporum tobira* leaves Thunb. (Apiales: Pittosporaceae) in the study population. It was of interest to examine whether olinscides exhibit characteristics of systemic insecticides that move through plants [52]. To assess this, in one group of leaves affected by the pest, the pest-free adaxial side of leaves was treated with olinscide Coccus-11, while in another group of leaves exposed to the pest, the abaxial side of leaves (where the pest was present) was treated as the control. On the abaxial side, Coccus-11 caused substantial mortality (85.3 ± 8.9%) compared to water-treated control group (χ^2^ = 76.4, *p* < 0.001, N = 140, df = 1). When applied to the adaxial side, Coccus-11 caused moderate mortality on the abaxial side residing pests (31.8 ± 7.1%) (χ^2^ = 11.6, *p* < 0.001, N = 133, df = 1) (Table 1).

The random (somewhat complementary) oligonucleotide A_2_C_3_G_3_T_3_-11 did not show a significant insecticidal effect compared to the water-treated control on either leaf surface on the fourth day. Thus, oligonucleotide insecticides based on unmodified antisense DNA do not exhibit pronounced systemic properties, and direct contact of the oligonucleotide insecticides with insect integument is necessary for substantial insecticidal effects.

### 2.5. Differential Gene Expression Analysis (DGE) After Contact Application of Coccus-11

On the fourth day, DGE of *C. hesperidum* in response to contact application of Coccus-11 was performed (Supplementary File). This time point was selected to capture the transition from the first (ribosome arrest and target rRNA hypercompensation) to the second (target rRNA degradation) phases of the DNAc mechanism, when the maximum number of proteins are involved in the process (Figure 3a). Particular attention was given to genes related to ribosomal proteins and ribosome biogenesis, as well as those involved in cellular energy production. Ribosome biogenesis and function are known to consume 60% of the total cellular energy [53], making it crucial to evaluate how these systems were functioning. Additionally, RNase H1 activity was evaluated due to its known role in degrading target RNA via complementary DNA [54]. DGE revealed that nearly all investigated ribosomal proteins of the 40S and 60S subunits were significantly upregulated, promoting new ribosome formation alongside hypercompensated rRNA. Major ribosome biogenesis proteins (NOP53, UTP30, NSA2, MAK21, BRX1, WDR12) were also upregulated (Table 2) [55].

Simultaneously, most of the investigated kinases (including mTOR, a serine/threonine protein kinase, a key player in ribosome biogenesis acting with RAPTOR via mTORC1) were downregulated [56], while mitochondrial ATP synthase and mitochondrial enzymes crucial for cellular energy production (e.g., phosphoenolpyruvate carboxykinase, cytochrome c oxidase, Acyl-CoA dehydrogenase, alcohol dehydrogenase, adenylate kinase, NADH dehydrogenase (ubiquinone), succinate-CoA ligase) were significantly upregulated [57,58]. This indicates an energy deficiency caused by oligonucleotide insecticide Coccus-11. Moreover, enzymes involved in lipid energy production [59] (e.g., triacylglycerol lipase (PNLIP), lysophospholipase III (LYPLA3), lysosomal acid lipase/cholesteryl ester hydrolase (LIPA), and secretory phospholipase A2 (SPLA2), PLA2G), were significantly upregulated. Meanwhile, crucial glycolytic enzymes [60,61] such as pyruvate kinase, aldolase, and phosphofructokinase-1 were downregulated, and none were upregulated, indicating a metabolic switch from carbohydrates- to lipids-based energy production due to its higher yield per unit mass. Importantly, RNase H1 was significantly upregulated (2.4-fold) during DNAc. RNase H1 cleaves RNA in RNA-DNA hybrids, including those formed with rRNA, and operates independently of the cell cycle [62]. Of note, crucial enzymes of RNA interference (DICER1, Argonaute 2, and DROSHA) were significantly downregulated (p < 0.01) in this experiment emphasizing fundamental difference in molecular mechanisms of DNAc and RNAi.

## 3. Discussion

### 3.1. Sequences of DNA Oligos Highly Complementary to rRNA (Olinscides)

**Figure 6:**
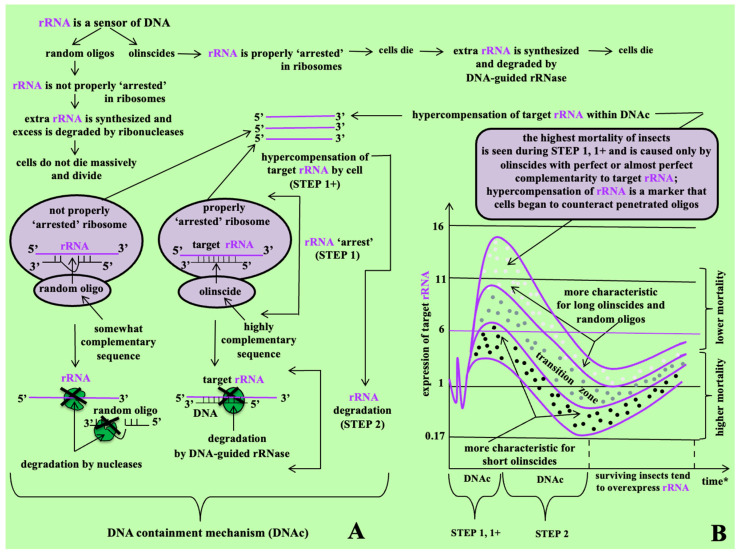
DNA containment mechanism (DNAc) of Sternorrhyncha representatives (**A**) and trends of target rRNA expression triggered by olinscides and random oligos during DNAc (**B**); *—time varies depending on distinct species and used oligos, but usually 2 steps (full cycle) of DNAc occur within 1–2 weeks; transition zone—can be detected less frequently for short olinscides, long olinscides, and random oligos.

In our study on sternorrhynchans, particularly *C. hesperidum*, we found that the DNAc mechanism consists of two steps and represents a previously unknown interplay between different types of DNA oligos (olinscides and random oligos) and rRNAs. This two-step DNAc mechanism has been clearly demonstrated in earlier studies involving oligonucleotide insecticides applied to *C. hesperidum* [16], *Trioza alacris* [13], *M. sanborni* [63], and also to *Aonidia lauri* and *Dynaspidiotus britannicus* [18]. Our current results further demonstrated that this mechanism operates not only for short 11-mer DNA sequences but also for long 56-mer DNA fragments. These findings enabled us to develop a general model of action for target-specific and random DNA oligonucleotides in sternorrhynchans, particularly *C. hesperidum* (Figure 6). The rRNA, which constitutes approximately 80% of total cellular RNA and plays critical roles in protein biosynthesis, apoptosis, DNA damage repair, serves as a potent sensor for exogenous DNA oligos. Therefore, regulation of rRNA synthesis is vital for cell function and survival [64,65]. The DNAc mechanism likely serves broader roles, possibly contributing to DNA repair, host–virus interactions, the cellular response to extracellular DNA, and endogenous regulation of rRNA expression, and may represent an evolutionarily conserved regulatory process in insects. The DNAc mechanism, as presently understood, likely represents just the visible part of a more complex set of cellular processes triggered by DNA oligos, particularly involving associated protein machinery.

In the first step of DNAc, an antisense DNA oligonucleotide (olinscide) binds complementarily to a mature rRNA and/or to 47S pre-rRNA. This binding blocks the normal function of mature ribosomes and disrupts the processing of 90S pre-ribosomes, leading to substantial insect mortality. In response, target rRNA hypercompensation occurs through the action of DNA-dependent RNA polymerase I (Pol I), which overproduces rRNA transcripts to compensate for the arrested function. Both the blocked ‘old’ rRNA within ribosomes and the newly synthesized rRNA and polycistronic transcripts (e.g., 47S pre-rRNA) can be detected via RT-PCR, demonstrating the hypercompensation process. However, the fine details of molecular architecture of this step remains unresolved. In the second step of DNAc, DNA-guided ribonucleases—such as RNase H1—cleave the targeted rRNA, causing a substantial reduction in its abundance. This step is also associated with high insect mortality. Both short and long olinscides either maintain low rRNA levels (e.g., Coccus-11 and Coccus_(−2)_-11, compared to short random DNA oligos) or significantly reduce rRNA levels after an initial hypercompensation peak (e.g., Coccus56_antisense_ and dsCoccus56, compared to long random DNA oligos), due to DNA-guided rRNase activity (such as RNase H1). These enzymes, such as RNase H1, are likely able to protect their unmodified DNA guides from DNase-mediated degradation.

Our study provides important insights into the fine details of the DNAc mechanism. Notably, treatment with the oligonucleotide insecticide Coccus-11 led to increased expression of ribosomal proteins, promoting ribosome biogenesis along with hypercompensated rRNA. Concurrently, ATP synthesis increased in mitochondria, primarily through lipid degradation. However, this ultimately resulted in a ‘kinase disaster’ as ATP depletion caused by rRNA synthesis and ribosome biogenesis led to widespread downregulation of kinases. Since kinases are involved in about 50% of all cellular reactions and play essential roles in cellular signaling and regulation via phosphorylation, their inactivity likely contributed to cellular exhaustion and insect death [66,67]. These findings support the central DNAc mechanism: initial rRNA hypercompensation followed by rRNA degradation mediated by DNA-guided rRNases, such as RNase H1, which was significantly upregulated in our study. Future investigations should explore other enzymatic systems’ responses to oligonucleotide insecticides and examine how fine details of the DNAc mechanism changes when targeting mitochondrial rRNAs or internal transcribed spacer (ITS) regions of pre-rRNA.

**Figure 7:**
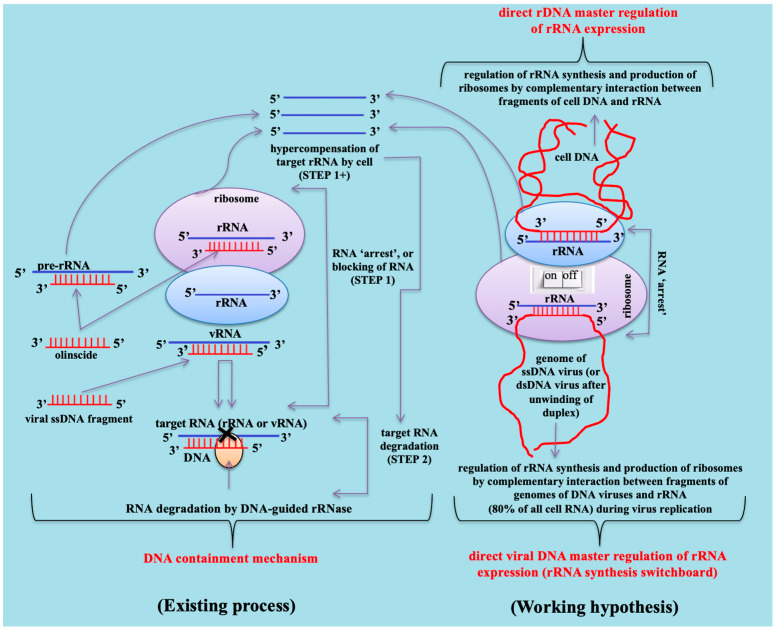
Mode of action of oligonucleotide insecticides, viral single-stranded DNA fragments, regulation of rRNA synthesis via direct rDNA transcription master regulation, and direct viral DNA master regulation of rRNA expression based on DNA containment mechanism.

As a working hypothesis, based on obtained results, DNA may act not only as a template for rRNA synthesis but also as a direct regulator of rRNA expression. The DNAc mechanism, particularly the hypercompensation phase (STEP 1+) (Figure 6), may play an important role in the regulation of rRNA by endogenous cell DNA (direct rDNA transcription control) and by viral DNA (rRNA switchboard mechanism) [11]. It may also serve as part of the innate immune defense against single-stranded DNA (ssDNA) viruses, for which hemipteran insects are major vectors [2,68], as well as against DNA viruses that naturally infect these insects [50]. Insect nucleases may cleave invader DNA, producing ssDNA oligos that subsequently guide the degradation of target viral RNAs [18] (Figure 7). Our results with unmodified DNA oligonucleotides, both single- and double-stranded, provide a compelling model for this process and open up a new dimension in the regulation of rRNA genes, which govern the expression of 80% of cell RNA.

### 3.2. Sequences of DNA Oligos Somewhat Complementary to rRNA (Random Oligos)

In the first step of the DNAc, a random DNA oligonucleotide with partial or imperfect complementarity to target rRNA weakly binds to mature rRNA and/or 47S pre-rRNA. As a result, it does not effectively block the normal functioning of mature ribosomes or the processing of 90S pre-ribosomes. In this case, hypercompensation of target rRNA by DNA-dependent RNA polymerase is likely a generalized cellular response to perceived DNA damage caused by the presence of the foreign DNA oligo. Insect cells respond by boosting protein biosynthesis, possibly through mechanisms that reorganize DNA damage repair factors via pre-rRNA during processes like meiosis or the DNA damage response [69]. Both weakly arrested ‘old’ rRNA in ribosomes and ‘newly’ synthesized rRNA transcripts (including 47S pre-rRNA) can be detected by RT-PCR, confirming the occurrence of rRNA hypercompensation. In the second step of DNAc with random oligos, the excess rRNAs are gradually cleaved by cellular ribonucleases, while the random DNA oligos are degraded by deoxyribonucleases. Given that rRNA has a typical cellular half-life of 3–5 days [47], a modest 2–3-fold decrease in rRNA concentration was observed between days 6 and 9. However, this was not accompanied by significant pest mortality in groups treated with DNA oligos. This suggests that imperfectly complementary oligos do not efficiently recruit DNA-guided rRNases, such as RNase H1, and the cells ultimately restore homeostasis by degrading the foreign oligos.

In general, random oligonucleotides tend to induce mild cellular responses (Figure 5b—A_2_C_3_G_3_T_3_-11), such as increased cell proliferation and cell volume, rather than cell death [16,70]. Both short and long single-stranded random oligos may trigger mild rRNA hypercompensation, but do not lead to lethal cellular outcomes. As the number of interacting biomolecules increases, nonspecific interactions become inevitable. To commit to a death signal, the cell requires a clear and strong trigger: a DNA oligo with perfect or near-perfect complementarity to rRNA. All weaker signals from random oligos are regulated by homeostatic mechanisms. Thus, only olinscides—DNA oligos with high sequence complementarity to target rRNA—induce significant pest mortality [18]. These findings open up promising new avenues for highly selective and effective insect pest control, while also revealing a novel regulatory mechanism underlying the biogenesis of the cell’s most abundant RNA-rRNA.

### 3.3. Horizons of Fundamental Understanding and Practical Application of DNAc Mechanism

In our view, closely related members of the Paraneoptera superorder, especially insects that are susceptible to DNA viruses or involved in transmitting plant DNA viruses, are most vulnerable to oligonucleotide insecticides. This vulnerability likely stems from their possession of a natural DNAc defense mechanism against DNA viruses, which enables them to recognize and degrade viral mRNAs [18]. Conversely, DNA viruses may also exploit DNAc mechanism, specifically rRNA hypercompensation, to boost rRNA production and ribosome biogenesis, thereby increasing the cell’s capacity for viral replication. Emerging genomic data suggests that many viral genomes contain sequences homologous to ribosomal RNA [71]. Furthermore, complementary sequences within host or viral DNA may regulate rRNA expression by forming DNA-rRNA duplexes that initiate the DNAc mechanism. Importantly, neither cell DNA nor viral DNA are at risk of degradation when they serve as guides for DNA-guided rRNases, such as RNase H1. Meanwhile, rRNA expression can be either upregulated or downregulated by complementary DNA sequences, making DNA a potential direct regulator of rRNA biogenesis. Our findings with unmodified DNA oligos reveal an entirely new layer of gene regulation affecting rRNA genes, which govern roughly 80% of the total cellular RNA pool.

One unresolved question is why long olinscides induce a more pronounced rRNA hypercompensation response than long random oligos, prior to the degradation phase of DNA-guided rRNases. The precise architecture of this response remains unknown. It is hypothesized that specific rRNA-sensing proteins monitor rRNA functionality by interacting directly with its structure [49,50], and some may act as these sensors. When a highly complementary DNA–rRNA duplex forms, these proteins may dissociate from the rRNA and relay a signal to initiate rDNA transcription. The greater the number of protein–RNA dissociations, the stronger the hypercompensatory response. Based on experimental observations from day 6 of rRNA hypercompensation (on which there is an approximately 9:1 ratio between Coccus56_antisense_ and water-treated control, Figure 4 and Figure 5), it is estimated that each such sensing protein will interact with 6–7 nucleotides (for dsCoccus-56, obviously after unwinding). This threshold may explain why partially complementary DNA oligos also trigger rRNA hypercompensation: even a short stretch of 6–7 matching bases may be sufficient to activate the response. All tested DNA oligos in this study meet this threshold when compared with the *C. hesperidum* 28S rRNA (via GenBank BLAST analysis). Thus, rRNA appears to function as a molecular sensor that detects both highly and partially complementary DNA sequences, with rRNA hypercompensation acting as one of the first cellular responses. This may explain why Coccus56_sense,_ while not producing strong hypercompensation, still causes significant pest mortality: it forms enough of a base pairing to interfere with normal interactions between 28S rRNA and ribosomal proteins. Notably, short 11-mer olinscides induced 5.76-fold lower hypercompensation compared to 56-mer olinscides, aligning well with their shorter length (which is 5.1-fold shorter), further supporting the rRNA-sensing protein hypothesis. Thus, the longer the olinscide, the stronger the rRNA hypercompensation.

Moreover, long olinscides may serve as models **for the natural interaction between host rRNA and DNA viruses that** infect hemipterans [72]. Antisense sequences like Coccus56_antisense_ and dsCoccus56 induced 2.36-fold higher hypercompensation of 28S rRNA compared to Coccus56_sense_ and the random sequence (ACGT)_14_-56, suggesting a potential strategy employed by viruses to co-opt host machinery. This phenomenon could be used by DNA viruses. It is obvious that DNA viruses can also take advantage of the hypercompensation of rRNAs observed during the DNA containment mechanism in order to increase the number of rRNAs for extra ribosomes necessary for their replication [73]. Bioinformatics evidence supports widespread viral hijacking of host genes [71,74], including lateral transfer of rRNA gene fragments for enhanced metabolic advantage during infection [75]. Since rRNA represents ~80% of total RNA and ribosome maintenance consumes more than 60% of the cellular energy spent on the production and maintenance of ribosomes [53,76], viruses likely evolved to manipulate rRNA synthesis efficiently. Most DNA viruses replicate in the nucleus and interact with the nucleolus, the site of ribosome biogenesis [24,77]. It is plausible that specific complementary viral DNA fragments function as **rRNA synthesis switches**, turning rRNA transcription ON or OFF to optimize host resource use during infection [17]. This simple ON/OFF regulation based on complementary base pairing could serve as a low-energy mechanism for viral control of host ribosome production. Importantly, our findings related to antisense-unmodified DNA show that even endogenous cell DNA may regulate rRNA expression in a similar fashion. Thus, beyond serving as the template for rRNA synthesis, cell DNA may also be a direct regulator of rRNA gene expression. These findings substantially alter our current understanding of rRNA biogenesis and gene regulation.

The implementation of the CUADb-based ‘genetic zipper’ method in crop protection could lead to a highly adaptable platform for designing oligonucleotide insecticides in response to genetic changes of pests during microevolution. The primary goal—safeguarding crop yields while minimizing the environmental impact—remains central to this approach. According to our latest projections, this method is also economically feasible for large-scale agricultural deployment. Chemical insecticides remain dominant in pest management [78,79,80,81,82], and pests are gradually developing resistance driven by natural selection via random mutations; this has been a major concern since the mid-20th century [8,78,83,84,85,86,87,88]. Antisense technologies such as RNAi, CUADb, and CRISPR/Cas offer solutions by targeting conserved gene regions and enabling rapid adaptation to resistance. CUADb and dsRNA technology, possessing different and potent molecular mechanisms, DNAc and RNAi, are especially promising as next-generation bioinsecticides, given their biodegradability, target specificity, and minimal environmental footprint [11,14,89,90,91,92,93,94,95,96,97,98,99], while CRISPR/Cas, more suited for genetic control of pest populations [100,101,102,103,104,105,106,107,108,109], may also complement these approaches. Together, these cutting-edge antisense technologies present a powerful toolkit for sustainable pest control. The key challenge ahead lies in choosing the optimal strategy for each specific pest, crop, and ecological context.

## 4. Materials and Methods

### 4.1. Origin of C. hesperidum L.

The *C. hesperidum* larvae were identified on *P. tobira* plants in the Nikita Botanical Garden (Yalta, Republic of Crimea, 44°30′41.9′′ N, 34°13′57.3′′ E) and used for the experiments. Treatments were applied to *P. tobira* plants using a hand-held sprayer with an aqueous solution of oligonucleotides (100 mg/L). The application rate was 1 mg of DNA in 10 mL of solution per m^2^ of foliage infested with the pest. Oligonucleotide insecticides were directly applied to first- and second-instar larvae. To ensure full coverage, the sprayer angle was adjusted so that the oligonucleotides reached the entire leaf surface harboring the pests. Across eight replicates, approximately 10,400 larvae were treated in three independent experiments. Insect survival was recorded. For each replicate and variant, 20 *P. tobira* leaves were assessed. Mortality was calculated by dividing the number of dead individuals by the total number of individuals per leaf and multiplying by 100 to express the result as a percentage.

### 4.2. Sequences and Applied Short (Coccus-11; Coccus_(−2)_-11) and Long (dsCoccus-56; Coccus-56_antisense_) Olinscides

Oligonucleotide insecticide sequences were designed based on the 28S rRNA sequence of *Coccus hesperidum* (isolate S6A395) retrieved from the GenBank database (https://www.ncbi.nlm.nih.gov/nuccore/MT317022.1, accessed on 27 March 2024). Two short oligonucleotides were used: Coccus-11 (5′–CCA–TCT–TTC–GG–3′) and Coccus_(−2)_-11 (5′–CAC–CAT–CTT–TC–3′). The latter located two nucleotides downstream of the antisense region targeted by Coccus-11. Two longer sequences were also applied: Coccus-56_antisense_, (5′–CCA–TCT–TTC–GGG–TAC–CAG–CGT–GCA–CGC–TGT–AGG-TGC–GCC–CCA–GTT–CGT–CGA–CGG–TC–3′) and dsCoccus-56, its corresponding double-stranded form. All oligonucleotides were dissolved in nuclease-free water at a concentration of 100 ng/μL and applied to *P. tobira* leaves at a rate of 10 mL per m^2^. A control group treated with water alone was included for comparison.

### 4.3. Sequences and Applied Short (A_2_C_3_G_3_T_3_-11; CG-11) and Long (Coccus-56_sense_; (ACGT)_14_-56) Random Oligos

As controls, the following random oligonucleotide sequences were used: short oligos A_2_C_3_G_3_T_3_-11 (5′–AAC–CCG–GGT–TT–3′) and CG-11 (5′–CGC–CGC–CGC–CG–3′) and long oligos Coccus-56_sense_ (5′–GAC–CGT–CGA–CGA–ACT–GGG–GCG–CAC–CTA–CAG–CGT–GCA–CGC–TGG–TAC–CCG–AAA–GAT–GG–3′) and (ACGT)_14_-56 (5′–ACG–TAC–GTA–CGT–ACG–TAC–GTA–CGT–ACG–TAC–GTA–CGT–ACG–TAC–GTA–CGT–ACG–TAC–GT–3′). These oligonucleotides were dissolved in nuclease-free water at a concentration of 100 ng/μL and applied using a hand-sprayer to *P. tobira* leaves (mg of oligonucleotide per m^2^). A total of 10 mL of solution was sprayed per m^2^. A water-treated group served as an additional control.

### 4.4. Synthesis of Oligonucleotides

All unmodified oligonucleotides were synthesized using the ASM-800ET DNA synthesizer (BIOSSET, Novosibirsk, Russia) with standard phosphoramidite chemistry on UnyLinker 500 Å universal solid support (ChemGenes, Wilmington, NC, USA). Cleavage and deprotection were performed overnight at 55 °C using concentrated ammonia. The solution was filtered and evaporated using a vacuum rotary evaporator (Heidolph Instruments GmbH & Co. KG, Schwabach, Germany). The resulting dry product was dissolved in deionized water (Merck Millipore, Molsheim, France) and the concentration was determined using a NanoDrop Lite spectrophotometer (Thermo Fisher Scientific, Waltham, MA, USA) [13].

### 4.5. Evaluation of 28S rRNA Expression of C. hesperidum

Larvae were homogenized in 1.5 mL tubes using a pestle, and RNA was extracted using the ExtractRNA kit (Evrogen, Moscow, Russia) per the manufacturer’s protocol. Three independent biological replicates were prepared, each using 10 larvae per treatment group. RNA concentration and quality were measured with a NanoDrop spectrophotometer. Electrophoresis was performed in 1.5% agarose gel with TBE buffer (10 V/cm for 30 min), loading 5 μL of RNA per lane [110]. Reverse transcription was performed using 50 ng of total RNA with the reverse primer (5′–ACG–TCA–GAA–TCG–CTG–C–3′) and the FastStart Essential DNA Green Master Kit (Roche, Basel, Switzerland) at 40 °C for 60 min in a LightCycler^®^96. The qPCR reaction used 2 μL of cDNA with forward (5′–ACC–GTC–GAC–GAA–CTG–G–3′) and reverse (5′–ACG–TCA–GAA–TCG–CTG–C–3′) primers and FastStart SYBR Green MasterMix (Roche). The PCR conditions were as follows: 10 min initial denaturation at 95 °C; 30 cycles of 10 s at 95 °C, 15 s at 62 °C; and 14 s at 72 °C [111]. Reactions were run in triplicate. Melt curve analysis confirmed the specificity of amplification.

### 4.6. Histochemical Assay

Tissues were dehydrated and paraffin-embedded using a Logos microwave histoprocessor (Milestone, Sorisole, Italy). Sections that were 4 µm thick were stained with hematoxylin and eosin. The reagents used included 10% formalin, isopropyl alcohol, paraffin, o-xylene, and Biovitrum staining kits (St. Petersburg, Russia).

### 4.7. Differential Gene Expression (DGE) Analysis

RNA quality and quantity were assessed using a BioAnalyser and the RNA 6000 Nano Kit (Agilent Technologies, Santa Clara, CA, USA). PolyA RNA was isolated using the Dynabeads^®^ mRNA Purification Kit (Thermo Fisher Scientific, Waltham, MA, USA). Libraries were prepared using the NEBNext^®^ Ultra™ II RNA Library Prep Kit (New England Biolabs, Beverly, MA, USA). Library concentrations and fragment size were assessed using a Qubit fluorometer (Thermo Fisher Scientific, Waltham, MA, USA) and High-Sensitivity DNA Kit (Agilent Technologies, Santa Clara, CA, USA). Sequencing was performed on an Illumina HiSeq1500 (Illumina, San Diego, CA, USA), generating at least 10 million 50 nt reads per sample. Reads were aligned to the genome using STAR, and differential expression was analyzed using DESeq2 (Bioconductor, Seattle, DC, USA). Reference genome: ihCocHesp2.1 (https://www.ncbi.nlm.nih.gov/datasets/genome/GCA_964257065.1/) (accessed on 2 August 2025). DGE was performed in two biological replicates for both the Coccus-11-treated and water-treated control groups, with 100 larvae per replicate.

### 4.8. Statistical Analyses

The standard error of the mean (SE) was determined and analyzed using Student’s *t*-test to evaluate the significance of the difference in 28S rRNA concentration between control and experimental groups on the second, sixth, and ninth days. The non-parametric Pearson’s chi-squared test (χ^2^) with Yates’s correction was performed to evaluate the significance of the difference in mortality between control and experimental groups on the second, sixth, and ninth days. All of the above-mentioned calculations were performed using Prism 9 software (GraphPad Software Inc., Boston, MA, USA).

## 5. Conclusions

The results obtained using antisense and double-stranded DNA fragments allowed us to propose a general framework for the interaction between rRNA and exogenous DNA, based on DNAc. This mechanism holds significance both for practical applications—such as DNA-programmable insect pest control—and for advancing our fundamental understanding of cellular function, particularly the regulation of rRNA synthesis and ribosome biogenesis. The DNAc process involves two main steps: first, the target rRNA is ‘arrested,’ leading to its hypercompensation; second, the target rRNA undergoes degradation mediated by DNA-guided rRNases, such as RNase H1. The unmodified DNA fragments tested here serve as simple yet robust models for studying complementary interactions between exogenous DNA (including viral DNA) and rRNAs, revealing new and previously unexplored roles of nucleic acids in cellular processes. In this study, the insecticidal potential of short 11-mer antisense DNA oligos was investigated for controlling *C. hesperidum*, in comparison with longer 56-mer single-stranded and double-stranded DNA sequences, and the latter was found to be less efficient. The shorter oligos demonstrated higher efficacy. Moreover, shorter sequences offer advantages in terms of higher synthesis yield via the phosphoramidite method, resulting in greater product mass and reduced production costs. Oligonucleotide insecticides act primarily as contact insecticides rather than systemic ones. Despite lacking pronounced systemic activity, they are highly effective due to their precise targeting of essential rRNA sequences in the pest, ultimately leading to insect mortality. The data indicate that pests are unable to compensate for the consequences of action of short-oligonucleotide insecticides, culminating in ATP depletion and a ‘kinase disaster’ leading to cell death. We are at the threshold of a new era of insecticide development, where control agents can be designed like a construction set—assembled from nitrogenous bases and guided by the genomic sequences of insect pests. Oligonucleotide insecticides are beginning to fulfill a 90-year-old vision: the creation of highly selective, potent and structurally adaptable chemical insecticides capable of keeping pace with the microevolution of pest populations.

## Figures and Tables

**Table 1:** Mortality of *C. hesperidum* after application of water, random oligo A_2_C_3_G_3_T_3_-11, and olinscide Coccus-11 on the abaxial and adaxial sides of *P. tobira* leaves on the fourth day.

Adaxial Side of the Leaf			Abaxial Side of the Leaf		
Control	A_2_C_3_G_3_T_3_-11	Coccus-11	Control	A_2_C_3_G_3_T_3_-11	Coccus-11
7.2 ± 1.9%	7.9 ± 5.5%	31.8 ± 7.1% *	7.5 ± 2.4%	16.6 ± 6.8%	85.3± 8.9% *

*—significance of the difference compared to water-treated control, *p* < 0.05.

**Table 2:** Differential gene expression (DGE) analysis of *C. hesperidum* after contact application of Coccus-11 vs. water-treated control group on the fourth day; * difference in the DGE of each protein in Coccus-11 group vs. water-treated control corresponds to the represented *p*-value.

Protein	Expression (Upregulated or Downregulated)	*p*-Value *
**Ribosomal proteins**		
60S subunit of ribosome RP-L19e; RP-L8e; RP-LP1; RP-L6e; RP-L34e; RP-L10e; RP-L4e; RP-L27e; L13; RP-L32e; RP-L17e	upregulated	*p* < 0.05
40S subunit of ribosome RP-S18e; RP-S3Ae; RP-S8e; RP-S9e; RP-S11e; RP-S30e; RP-S3e; RP-S24e; RP-S15e; RP-S4e; RP-S21e	upregulated	*p* < 0.05
**Ribosome biogenesis proteins**		
NOP53; UTP30; NSA2; MAK21; BRX1; WDR12	upregulated	*p* < 0.04
**Proteins involved in regulation of ribosome biogenesis**		
mTOR; RAPTOR	downregulated	*p* < 0.01
**RNase H1**		
rnhA	upregulated	*p* < 0.001
**Glycolytic enzymes**		
Pyruvate kinase; pyruvate carboxylase; 6-phosphofructokinase 1	downregulated	*p* < 0.02
**Enzymes involved in production of energy from lipids**		
PNLIP; LYPLA3; LIPA; SPLA2; PLA2G	upregulated	*p* < 0.001
**Mitochondrial ATP synthase complex (complex V) proteins**		
ATPeF1B; ATPeF1G; ATPeF0D; ATPeF0E; ATPeF0F; ATPeF0F; ATPeFG; ATPeF0O	upregulated	*p* < 0.05
**Mitochondrial proteins involved in energy production**		
pckA; ACADS; ACADSB; GCDH, ACOX1, ADHFE1; subunits of cytochrome c oxidase (VIIc; COX4; Vb; COX5A; Via; VIc/VIIs; COX5B); subunits of NADH dehydrogenase (NDUFA2; NDUFB10; NDUFA13; NDUFS4; NDUFS5; NDUFA12; NDUFV3; NDUFAB1; NDUFV2; NDUFA5; NDUFS8; NDUFB4; NDUFS6; NDUFS7; NDUFA10; NDUFA7; NDUFB11; NDUFS3; NDUFA8; NDUFAF4; NDUFA11; NDUFB6)	upregulated	*p* < 0.05
**Kinases**		
PINK1; MAP2K4; PLK1; WEE1; TLK; ADRBK; MAP2K1; BUB1; FLT1; dgkA; AAK; PIP5K; TNK2; STK24; CDK12; EPS8; MUSK; SRPK1; STK11; MAPKAPK2; PDPK1; FRK	downregulated	*p* < 0.01
**RNAi pathway enzymes**		
DICER1, Argonaute 2, DROSHA	downregulated	*p* < 0.01

## Data Availability

The data presented in this study are openly available in [repository FigShare] at [https://doi.org/10.6084/m9.figshare.28938248] (accessed on 2 August 2025).

## Supplementary Materials

The following supporting information can be downloaded at: https://www.mdpi.com/article/10.3390/ijms26157530/s1.
